# Measuring Coping in Parents of Children with Disabilities: A Rasch Model Approach

**DOI:** 10.1371/journal.pone.0118189

**Published:** 2015-03-02

**Authors:** Vijaya K. Gothwal, Seelam Bharani, Shailaja P. Reddy

**Affiliations:** 1 Meera and L B Deshpande Centre for Sight Enhancement, L V Prasad Eye Institute, Hyderabad, India; 2 Bausch and Lomb School of Optometry, L V Prasad Eye Institute, Hyderabad, India; Merced, UNITED STATES

## Abstract

**Background:**

Parents of a child with disability must cope with greater demands than those living with a healthy child. Coping refers to a person’s cognitive or behavioral efforts to manage the demands of a stressful situation. The Coping Health Inventory for Parents (CHIP) is a well-recognized measure of coping among parents of chronically ill children and assesses different coping patterns using its three subscales. The purpose of this study was to provide further insights into the psychometric properties of the CHIP subscales in a sample of parents of children with disabilities.

**Methods:**

In this cross-sectional study, 220 parents (mean age, 33.4 years; 85% mothers) caring for a child with disability enrolled in special schools as well as in mainstream schools completed the 45-item CHIP. Rasch analysis was applied to the CHIP data and the psychometric performance of each of the three subscales was tested. Subscale revision was performed in the context of Rasch analysis statistics.

**Results:**

Response categories were not used as intended, necessitating combining categories, thereby reducing the number from 4 to 3. The subscale – ‘maintaining social support’ satisfied all the Rasch model expectations. Four item misfit the Rasch model in the subscale –maintaining family integration’, but their deletion resulted in a 15-item scale with items that fit the Rasch model well. The remaining subscale – ‘understanding the healthcare situation’ lacked adequate measurement precision (<2.0 logits).

**Conclusions:**

The current Rasch analyses add to the evidence of measurement properties of the CHIP and show that the two of its subscales (one original and the other revised) have good psychometric properties and work well to measure coping patterns in parents of children with disabilities. However the third subscale is limited by its inadequate measurement precision and requires more items.

## Introduction

Looking after a child with disability is challenging both physically and psychosocially given that it usually spans the course of a child’s life, exceeding typical child development needs and that parents as well as families are not at all prepared for it [1, 2]. As a part of the care giving responsibility, parents encounter a variety of challenges such as overcoming the disappointments attendant to the original diagnosis and the need to coordinate the child’s multifaceted medical, educational, and developmental interventions while balancing competing family needs [3]. Furthermore caring for a child with disability often requires additional physical, emotional, social, and financial resources [4]. It is also noted that having a child with disabilities affects not only the parents, but also siblings and the relationships among the family members [5]. In order to respond to the increasing demands of a stressful family situation and to confront these difficult situations, parents of children with disabilities develop coping strategies to reduce tension and anxiety, and thus adapt to their new situation.

Coping refers to a person’s cognitive or behavioral efforts to manage the demands of a stressful situation [6]. Previous research has also shown that many families of chronically disabled children succeed in adapting and functioning well and among various factors studied, the type of disability, parental personality, the age of the child when diagnosed, demographic variables such as parental educational level, gender and socioeconomic status have been found to be associated with parental coping and adjustment [7–12].

The Coping Health Inventory for Parents (CHIP) is among the popular instruments to measure parental coping patterns when parents have a child who is seriously and/or chronically ill [13–16]. It was developed and validated using classical test theory (CTT). The CHIP comprises three subscales (dimensions) measuring three different coping patterns: (a) maintaining family integration, co-operation, and an optimistic definition of the situation; (b) maintaining social support, self-esteem, and psychological stability; and (c) understanding the healthcare situation through communication with other parents and consultation with the healthcare team. Using CTT, each of the subscales has been shown to possess adequate internal reliability (Cronbach alpha = 0.79, 0.79, and 0.71 respectively) and validity [14]. Each item has four response categories on a Likert scale ranging from 0 to 3 (“not helpful”, “minimally helpful”, “moderately helpful” and “extremely helpful”). These values are added together for each subscale to produce a subscale score (Likert scoring). A major criticism of such a scoring method (and thus its interpretation) is that it is based on ordinal-level data [17, 18] in which the distance between response categories is assumed to be (and thus treated) equidistant. However this assumption is flawed given that the distance between response categories is unknown. For example, the distance between “not helpful” and “minimally helpful” may be different to the distance between “minimally helpful” and “moderately helpful”. This approach limits the interpretation of the subscale score because the difference between a score of 30 to 32 on an ordinal scale may not represent the same distance as a score between 32 and 34. This precludes mathematical operations such as calculation of change scores or effect sizes. Therefore, CTT provides outcome measures which are ordinal in nature precluding use of parametric statistical tests which nevertheless seem to be carried out frequently [19–22].

Over the past few years, research has shown that instruments that were developed using CTT could benefit substantially from modern approaches such as item response theory, specifically Rasch analysis [18, 23–25]. Compared to CTT, however, Rasch analysis (where items and persons can be scaled according to a series of response to items) provides greater insight into an instrument’s psychometric properties such as uniimensionality, an assessment of differential item functioning (DIF), and linear scaling properties [26, 27]. Unidimensionality refers to the extent to which the items within the scale measure a single construct [28] and is essential for valid summation of items, and linear measurement is an essential prerequisite for parametric analyses and meaningful calculation and interpretation of change scores. DIF refers to a form of item bias and occurs when items do not operate in the same way for different groups or subgroups (e.g. mothers and fathers) who otherwise have the value on the latent variable. This means that the items do not work in the same way when answered by mothers or fathers and their results would not be comparable [29, 30]. If the data fit the Rasch model well, the model then transforms an ordinal raw score into a linear, interval-level variable which improves sensitivity to change and, therefore, has advantages for outcomes research [31, 32]. There is growing interest in assessing coping using CHIP and the characteristics that may influence parental coping when caring for a child with disability or chronic illness [13, 33]. However as mentioned earlier the CHIP hasn’t undergone rigorous assessment of its measurement properties using a Rasch model approach. As a result, gaps remain in our knowledge whether CHIP has robust psychometric properties.

In this paper, we use Rasch analysis to explore the psychometric properties of the three CHIP subscales, specifically, the extent to which the three subscales form unidimensional scales with linear measurement properties. In the event that any of the subscales fail to meet the stringent requirements of the Rasch model, then we would consider re-engineering it to optimize its measurement properties (if possible) such that the resulting interval-level scores could be used in public health studies and in other areas of healthcare.

## Patients and Methods

### Study design and participants

Participants for this study comprised of parents of children with disabilities enrolled in special schools as well as in mainstream schools in the city of Hyderabad, South India. Hyderabad (the capital city of the newly formed South Indian state of Telangana) is advantageous for such a cross-sectional study as it contains the maximum number of special schools (approximately 36) for a total population of over 8 million, thereby making it one of the most representative Indian cities for such a cross-sectional study involving parents of (school age) children with disabilities. Participants were eligible if they were the parent who daily took care of a school age child with a disability, and could speak/understand English or a local language (Telugu).

Potential parents were contacted through the respective special schools in which their child was enrolled. Prior to data collection, the principal investigator established contacts with these schools and discussed the details with the school administrators who in turn discussed it with the parents of children enrolled in their respective schools. Following this initial briefing about the study, research assistants (RAs) contacted the parents (when they came to drop/pick their child from school) and invited them to participate. Following this, RAs provided instructions in completing the package to the consenting parents. To maximize recruitment rate, the RAs also recruited parents of children with visual disability (enrolled in either special school/mainstream school) and were provided low vision rehabilitation services at our centre during the same period. The package contained the 45-item CHIP and socio-demographic information, including parent’s and child’s age, gender, type of disability diagnosed in their child (if known, and if the parent was not aware of this then the data was collected from the child’s school), parent literacy, employment status, family system (nuclear or joint), number of children with disabilities, duration of disability, and approximate monthly family income (in Indian Rupees, INR). Parents were given the choice of either completing the package at their child’s school, at the centre or at home. Those parents who completed the package at home returned it to the school authorities, following which the RAs verified it for completeness and any missing data was completed over phone. In case of illiterate parents, trained research staff administered the CHIP to each parent in a face-to-face interview conducted in a quiet location identified at each child’s respective school and took approximately 15–20 minutes to complete.

### Ethics Statement

Ethical approval of the study was obtained from the Ethics Committee for Human Research at the L V Prasad Eye Institute, Hyderabad, India and all consenting participants provided written informed consent. The study adhered to the tenets of the Declaration of Helsinki.

### Coping Health Inventory for Parents (CHIP)

The 45-item CHIP was used to assess parents’ coping patterns they were using to manage family life when they had a disabled child [14] (Table 1).

**Table 1:** Item content of the 45-item Coping Health Inventory for Parents (CHIP).

**Item No.**	**Item Description**	**Subscale**
**1**	**Believing that my child (ren) will get better**	Maintaining family integration, co-operation, and an optimistic definition of the situation
**3**	**Doing things with my children**	
6	Building a closer relationship with my spouse	
8	Doing things with family members	
11	Believing that my child is getting the best medical care possible	
13	Doing things together as a family (involving all members of the family)	
16	Getting other members of the family to help with chores and tasks at home	
18	Believing that the medical center/hospital has my family’s best interest in mind	
21	Being able to get away from the home care tasks and responsibilities for some relief	
23	Eating	
26	Purchasing gifts for myself and/or other family members	
**28**	**Working, outside employment**	
**31**	**Talking to someone (not professional counselor/doctor) about how I feel**	
36	Building close relationships with people	
38	Talking with other parents in the same type of situation and learning about their experiences	
41	Reading more about the medical problem which concerns me	
43	Being sure prescribed medical treatments for child(ren) are carried out at home on a daily basis	
44	Talking with other individuals/parents in my same situation	
45	Talking with the doctor about my concerns about my child (ren) with the medical condition	
2	Investing myself in my children	Maintaining social support, self-esteem, and psychological stability
4	Believing that things will always work out	
7	Talking over personal feelings and concerns with spouse	
9	Believing in god	
12	Trying to maintain family stability	
14	Trusting my spouse (or former spouse) to help support me and my child (ren)	
17	Having my child with the medical condition seen at the clinic/hospital on a regular basis	
19	Encouraging child(ren) with medical condition to be more independent	
22	Getting away by myself	
24	Sleeping	
27	Concentrating on hobbies (art, music, jogging, etc.)	
29	Becoming more self-reliant and independent	
32	Engaging in relationships and friendships which help me to feel important and appreciated	
33	Entertaining friends in our home	
34	Investing time and energy in my job	
37	Developing myself as a person	
39	Talking with the medical staff (nurses, social worker, etc.) when we visit the medical centre	
42	Explaining our family situation to friends and neighbors so they will understand	
5	Telling myself that I have many things I should be thankful for	Understanding the healthcare situation through communication with other parents and consultation with the healthcare team
10	Taking good care of all the medical equipment at home	
15	Showing that I am strong	
20	Involvement in social activities (parties, etc.) with friends	
25	Allowing myself to get angry	
30	Keeping myself in shape and well-groomed	
35	Going out with my spouse on a regular basis	
40	Reading about how other persons in my situation handle things	

Framing question for all the items is “for each coping behavior you used, please record how helpful it was”.

Items in bold represent misfitting items.

Parents were asked to record how helpful each coping strategy was in their family situation on a Likert scale of 0–3, with 0 indicating ‘not helpful’ and 3 indicating ‘extremely helpful.’ As stated earlier, the instrument measures three coping patterns via three subscales. The first subscale consists of 19 items and measures “maintaining family integration, co-operation, and an optimistic definition of the situation” that focuses on strengthening family life and relationships and the parents’ outlook on life with a chronically ill child (e.g. ‘trusting my spouse to help support me and my child’). The second subscale consists of 18 items and measures “maintaining social support, self-esteem, and psychological stability” that focuses on parents’ efforts to develop relationships with others, engage in activities that enhance feelings of individual identity and self-worth plus strategies to manage psychological tensions and pressures (e.g. ‘engaging in relationships and friendships which help me feel important and appreciated’). The third subscale consists of eight items that measure “understanding the healthcare situation through communication with other parents and consultation with the healthcare team” and are directed at the parents’ relationships with healthcare professionals and other parents of chronically ill children (e.g. ‘talking with the medical staff when we visit the medical clinic’). Instruments require adaptation and translation for use in another country that has both a different culture and language. Consequently, we used standard guidelines for translation of instruments and performed a forward-backward translation, including a cognitive de-briefing in a representative sample, using a multistage iterative process, prior to adapting it for our local use [34]. Higher subscale scores imply greater help of that particular coping pattern.

### Rasch analysis

Rasch analysis was conducted according to the Andrich rating scale model for polytomous data (i.e. multiple response options for an item) [35] using Winsteps software, version 3.68 [36]. Rasch analysis was performed in a series of steps that included the following: (1) assessment of rating scale (i.e., if higher response categories represented greater use of that particular coping pattern), (2) measurement precision (represented by person separation reliability, PSR; minimum acceptable value of 0.8 [37]), (3) unidimensionality (i.e., if all the items contribute and measure a single underlying construct of coping measured by infit mean square statistic with acceptable range of 0.7–1.3, and also by principal components analysis of residuals, PCA whereby an unexplained variance explained by first contrast (eigenvalue size) less than 3.0 was considered good [38, 39]), (4) if item endorsability matches the parent’s coping pattern, represented by targeting (ideal <0.5 logits), and (5) DIF—if the data fit the Rasch model, Rasch analysis allows detection of differences in item difficulties between different groups within a sample [37]. We selected the DIF variables *a priori* in the present study. DIF was investigated for parent gender, disability type (any disability other than visual or visual plus any other disability). DIF was considered to be notable if > 1.0 logits [40, 41]. We considered the fundamental requirement of minimum PSI of 2.0 for each subscale to be reliable.

Descriptive data was analyzed using the Statistics Package for the Social Sciences (SPSS, version 16.0; SPSS Inc., Chicago, IL).

## Results

### Participant characteristics

Two hundred and seventy eligible parents were contacted and invited to participate in the study. Of these, 50 declined to participate due to following reasons: (a) 28 cited lack of time; (b) 15 decided not to take part without giving any reasons; and (c) 7 did not return the package. The final sample for this study consisted of 220 parents of children with disabilities (response rate, 81%). The majority of the participants were mothers (85%), literate (80%), married (95%) and self-administered the CHIP (78%). The mean ± SD age of the parents was 33.4 ± 7.1 years (range, 20–62 years). Eighty percent of the parents had completed secondary level or higher degree education and 69% were not working. Demographic data of the participants is summarized in Table 2. A little over half of the children (127, 58%) had either visual or hearing disability. Specific type of disability is shown in Table 2.

**Table 2:** Sociodemographic characteristics of parents of children with disabilities who responded to the Coping Health Inventory for Parents (n = 220).

Characteristic	Value
Parent	
Age (years)	
Mean ± SD	33.4 ± 7.1
Range	20–62
Gender, n (%)	
Female	187 (85)
Families with > 1 disabled child, n (%)	35 (15)
Education, n (%)	
Illiterate/elementary school only	44 (20)
High school/college/university	176 (80)
Marital status, n (%)	
Married	209 (95)
Divorced/widowed	11 (5)
Employment status, n (%)	
Not working	152 (69)
Family structure, n (%)	
Nuclear	133 (60)
Joint	87 (40)
Household income (INR) *, n (%)	
≤10,000	139 (63)
>10,000	81 (37)
Care recipient (Child)	
Age (years)	
Mean ± SD	9.5 ± 4.2
Range	1–16
Gender, n (%)	
Male	143 (65)
Type of disability	
Visual	66 (30)
Hearing	61 (28)
Cerebral palsy	15 (7)
Autism	44 (20)
Genetic syndromes	17 (8)
Global developmental disabilities	11 (5)
Attention deficit hyperactivity disorder	4 (2)
Unknown	2 (1)
Time elapsed since diagnosis of child’s disability (years)	
Mean ± SD	7.5 ± 4.0
Range	1–15

INR—Indian rupees

## Rasch analysis

### Rating scale assessment

**Fig 1:**
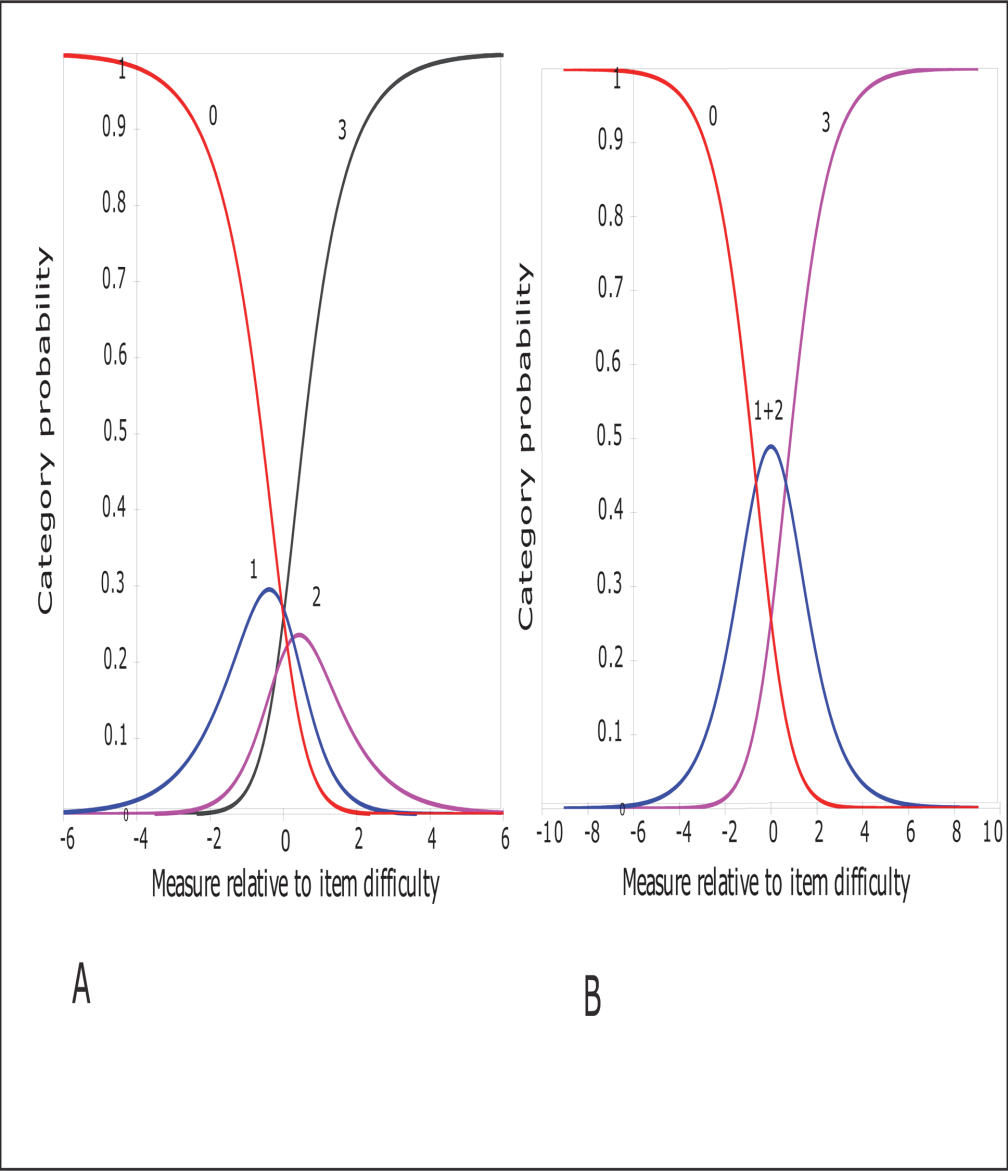
Rasch model category probability curves for all items together in the CHIP showing the likelihood that a participant with a particular coping ability will select a category. The scale (*x*-axis) from +6 to-6 symbolizes the latent trait of coping and the *y*-axis represents the probability of category being selected. Response categories: 0 “not helpful”, 1 “minimally helpful”, 2 “moderately helpful”, and 3 “extremely helpful”. For any given point along this scale, the category most likely to be chosen by a participant is shown by the category curve with the highest probability. At no point, was category 2 the most likely to be chosen, resulting in disordered thresholds (A). Thresholds represent boundaries along the scale where the probability of a response category being chosen changes from one to the next. However, combining categories 1 and 2, and thereby reducing the number of categories from 4 to 3 repaired the disordered thresholds for the category probability curves (B).

Participants did not use the response categories as intended. Categories that function well should have ordered structure calibration thresholds. This indicates that each category has a distinct probability of being chosen more than any other category for a particular item endorsability. However, this was not the case. Fig. 1A shows the category probability curves (CPC) which illustrate the range of coping patterns for which each of the 4 response categories were most likely to be chosen. It can be seen that at no point on the logit scale was the probability of responding to category 2 “moderately helpful” greater than the probability of responding to category 1 (“minimally helpful”) or category 3 (“extremely helpful”). Therefore, this response category does not function as expected and resulted in disordered thresholds. This category could be either combined with category 3 or 1. However, we chose the category combination that provided the best targeting and PSR. Consequently, we rescored the response categories by collapsing category 2 and 1 into a single new category “somewhat helpful” (0112), which improved threshold ordering for this rating scale (Fig. 1B). This reduced the number of categories from 4 to 3 and all thresholds were ordered.

### Overall performance of maintaining family integration, co-operation, and an optimistic definition of the situation subscale

Of the 19 items, four (21%) misfit the Rasch model and their infit MnSq values were all >1.3 (Tables 1 and 3).

**Table 3:** Overall performance of the CHIP Subscales in parents of children with disabilities.

Parameter	Ideal values	Versions of CHIP Subscales			
		MaintainingFamily integration		Maintaining social support	Understanding health care situation
		Original	Revised*		
Number of items	-	19	15	18	8
No. of misfitting items	0	4	0	0	0
Person separation	≥ 2.0	2.18	2.24	2.02	1.27
Reliability	≥ 0.80	0.83	0.84	0.80	0.62
Mean item location	0	0	0	0	0
Mean person location	0	0.51	0.65	0.44	-0.01
Principal components analysis (eigenvalue)	≤3.0	2.5	2.3	2.7	1.7
Differential item functioning, DIF (Number of items with notable DIF, >1.0 logits)	0	0	0	0	0

CHIP- Coping Health Inventory for Parents.

* Misfitting items were deleted iteratively and the final 15-item revised version is only shown here. See text for details (results section).

The misfitting items did not appear to be in tandem with the remaining items in the measurement of the underlying construct. Consequently, these four items were removed iteratively, resulting in a 15-item subscale (Table 3). Removal of these items improved the fit of the scale to the Rasch model and the infit MnSq was within the acceptable range for the remaining 15 items. Thus the 15-item “maintaining family integration, co-operation, and an optimistic definition of the situation” subscale possessed a PSR 0.84), implying adequate reliability (i.e. measurement precision). Targeting was 0.65 logits, suggesting that the items were matched well with the participant’s coping patterns. The unexplained variance explained by the first contrast was 2.3 eigenvalue units, indicating unidimensionality. No item displayed notable DIF (Table 3). Rather than discard the four deleted items, we assessed if they could be used to form a valid subscale. However they could not form a valid measure.

### Overall performance of maintaining social support, self-esteem, and psychological stability subscale

All the 18 items fit the Rasch model well and the PSI was 2.02 (reliability = 0.80), implying adequate measurement precision (Table 3). Targeting was optimal (0.44 logits). The unexplained variance explained by the first contrast was 2.7 eigenvalue units that satisfied the requirements for unidimensionality. No item displayed notable DIF.

### Overall performance of understanding the healthcare situation through communication with other parents and consultation with the healthcare team subscale

All the eight items fit the Rasch model well. However the PSI was 1.27 (reliability = 0.62) which is much lower than the minimum accepted value of 2.0, rendering it dysfunctional (Table 3). The unexplained variance explained by the first contrast was 1.7 eigenvalue units. None of the items displayed notable DIF.

## Discussion

This study provides new insights into the measurement psychometric properties of the subscales of CHIP in parents of children with disabilities in India. Using Rasch analysis, our results have demonstrated that the two revised subscales (“maintaining family integration, co-operation, and an optimistic definition of the situation subscale”, and “maintaining social support, self-esteem, and psychological stability”) of CHIP are unidimensional in our sample. However the subscale “understanding the healthcare situation through communication with other parents and consultation with the healthcare team” appears to be affected by inadequate measurement precision, thereby, rendering it dysfunctional in our population. If our revised response category format for the two functional subscales of CHIP is confirmed in an independent study, then this would have two important implications. Firstly, the use of summary or total subscale scores is justified for the two CHIP subscales (“maintaining family integration” and “maintaining social support”) and secondly, raw subscale scores can easily and legitimately be transformed into interval-scale estimates for these two subscales. Hence, researchers intending to calculate change scores or use parametric statistics can be confident that the transformed data satisfy this criterion [33].

The two CHIP subscales (“maintaining family integration” and “maintaining social support”) constitute a unidimensional hierarchical scale in which the individual item scores can now justifiably be summed to obtain a reliable summary index of parental coping patterns. Furthermore, the fact that both the subscales were unidimensional indicated that all the items in the respective subscales were in tandem regarding their contribution to the underlying construct (i.e., coping). However we used a lenient criterion in our analysis of unidimensionality given that we were re-validating a legacy instrument (versus developing a new instrument), so it is plausible that there is still some amount of multidimensionality in this subscale. Nonetheless, we do not believe this should be interpreted as a central critique of the two functional subscales of CHIP. As stated earlier, unidimensionality is an important prerequisite for summating any set of Likert-style items [42, 43] commonly seen in instruments used in health care, and therefore, constitutes an important advantage if meaningful measurement is to be obtained [43, 44]. Both the subscales demonstrated high measurement precision (high PSR) suggesting that the parameter estimates obtained in the present study are reproducible and useful for differentiating three groups of participants based on their performance. Both these subscales of CHIP can either be used alone or together to assess two separate coping patterns in parents of children with disabilities in a different cultural setting, i.e., among non-English (e.g., local Indian language) speaking populations.

Rasch analysis revealed the presence of four misfitting items (‘believing that my children will get better’, ‘doing things with my children’, ‘working, outside employment’, and ‘talking to someone about how I feel’) in one of the subscales (“maintaining family integration”‘) in this study. However the item misfit was relatively small with largest misfit being an item with infit MnSq, 1.46 (‘talking to someone about how I feel’). Item misfit indicates that the participants responded to these items differently than what the Rasch model expected. Unexpected responses or item misfit can occur for several reasons such as poorly constructed items, ambiguous wording, etc. In the present study, the reason for the four items to misfit (i.e. underfit) was perhaps because these items appear to be measuring another construct from the remaining items. Their deletion also resulted in a reduction in the length of the subscale which in turn reduces respondent burden.

It is also interesting to note that the rating scale required revisions (Fig. 1 A and B). The application of Rasch analysis to the CHIP has allowed greater scrutiny of the performance of the rating scale that would not have been possible with the traditional approach to instrument development (i.e., CTT). The response categories proposed for the original CHIP (English version) were found unsuitable for use in the Indian parents of children with disabilities. Our participants found it difficult to distinguish between the two intermediate categories—‘minimally helpful’ and ‘moderately helpful’. These categories were underutilized (used only 17% of the times) as compared to 40% for the end categories. Furthermore, the distance between these two categories was not sufficiently different and was 0.29 logits (which is less than the recommendation by Linacre of at least 1.4 logits apart [45]). Taken together, these findings suggested that we combine the intermediate categories so we performed post-hoc category re-organization to achieve optimal functioning of the rating scale, and we found a revised three-category rating scale (‘not helpful’, ‘somewhat helpful’, ‘extremely helpful’) to be adequate for the items as compared to the originally proposed four-category scale. While threshold disordering can be visualized on the CPC during Rasch analysis indicating the need for category re-organization, it is also important to note that it perhaps makes more sense and is less confusing to the participants when subtle differences such as between ‘minimal’ and ‘moderate’ do not exist in a response category format; rather such nearby categories should be combined and presented as a single category. More importantly, such reduction in number of categories also reduces the cognitive load on the respondent and makes it easier for them to complete the questionnaire. The need for collapsing adjacent categories and the resultant category re-organization is not uncommon in instruments with many response options, or when the labeling of options is too similar to each other, which can be confusing or open to misinterpretation. Our finding is consistent with other instruments, for example, vision-specific instruments that have benefitted from a shortening of their ratings scales following Rasch analysis. [18, 46–48]Issues with response categories can occur when the labeling of the response options is ambiguous or too many response options have been included. However, our findings of category re-organization may be a function of the distinct study population and not the measure itself. The need for such post-hoc category revision has been reported for other instruments that have been re-validated using Rasch analysis, for example, visual disability instruments, which have found that the participants are not always able to distinguish between finer increments (for example, between ‘more difficult’ and ‘a lot more difficult’) in response options [18, 49]. However, problems with rating scale structure of the Indian CHIP may be influenced by the relatively small sample used in this study and will need to be verified in larger and broader samples before specific recommendations can be made. Ideally this would include the administration of the original and revised versions of the CHIP scoring to the same people to compare their validity”.

**Fig 2:**
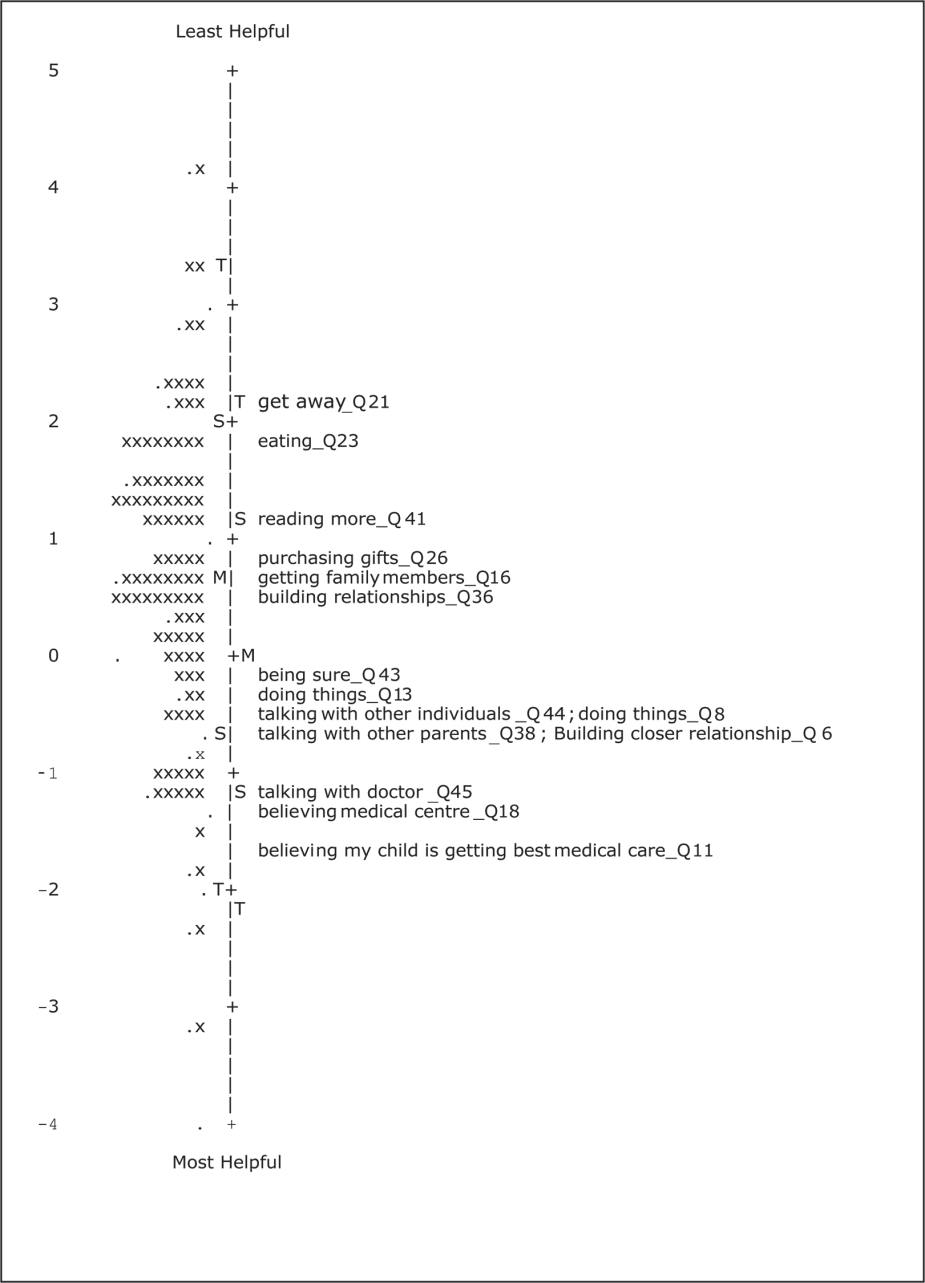
Person-item map for the Rasch-revised 15-item ‘maintaining family integration, co-operation, and an optimistic definition of the situation’ subscale of the Coping Health Inventory for Parents (n = 220). Participants are located on the left of the dashed line (represented by ‘x’) and participants with better coping ability are located at the top of the map. Items (i.e., coping patterns) are on the right of the dashed line with those considered to be least helpful located toward the top of the map. Each ‘x’ and “.” represent two and one participants respectively. Alongside each item is also indicated its abridged description and number as in the 45-item original CHIP. The complete description of items can be found in Table 2 in the text. M, mean; S, 1 SD from the mean; T, 2 SD from the mean.

Rasch analysis places the items and persons along the same scale, enabling simultaneous comparison of item endorsability and person’s coping patterns (Fig. 2). This feature is lacking in the CTT methodology [50, 51]. For a valid measurement, in addition to acceptable measurement precision, it must possess certain attributes, including adequate spread along the dimension of measurement, and negligible floor and ceiling effects [17]. While the subscale—“maintaining social support” had excellent targeting (0.44 logits), the items in the subscale—“maintaining family integration” were also reasonably well targeted to the participant’s coping patterns (0.65 logits). Furthermore, these two subscales did not demonstrate any DIF with regard to parent gender and child’s type of disability suggesting that it performs equally across mothers and fathers, and across different types of disability.

By comparison, the subscale—“understanding the healthcare situation” was dysfunctional primarily because of insufficient items (eight as compared to 19 and 18 in the other two subscales) resulting in poor person separation reliability. However, the low reliability is inconsistent with the original development study that reported relatively high reliability (0.71) for this subscale, albeit using the traditional measure, Cronbach alpha [14]. Nonetheless, Cronbach alpha is used as a reliability coefficient to represent the unidimensionality of an instrument, often exaggerated by the number of items in the instrument [42]. This limitation highlights the need for use of Rasch analysis either in the development [17, 37, 38, 52] or in the re-validation phase of the instruments [23, 24, 48, 53, 54]. As noted earlier, reliability and unidimensionality are fundamental to the Rasch models [17, 44]. Although this subscale lacked reliability, it was unidimensional. However, unidimensionality in the absence of reliability would be of limited value because we may be measuring too coarsely. That is, this subscale cannot discriminate among participants with varying coping patterns in terms of “understanding healthcare situation” effectively. All it can do is to separate the participants into those who consider this coping pattern as ‘not helpful’ and ‘extremely useful’ category. Such low measurement precision limits the usefulness of the subscale in the clinic as it does not help supplement the results of a clinical evaluation.

We acknowledge that our study has three important limitations. First, our study included a slightly higher proportion of women (mothers), and literate parents belonging to urban areas. However, this should not compromise the calibration of the instrument. Unlike the calibration of the instrument in the traditional test design which are dependent upon the sample, Rasch analysis allows relatively sample-free test calibration [55]. Second, the data in the present study were collected from those parents whose children were attending school, so it is unknown whether the results can be generalized to parents whose child (ren) with disability do not attend school, especially those residing in rural areas. Hence future studies should apply the CHIP to parents of children with disabilities who are out of school (especially in rural areas) and to evaluate whether DIF exists among such parents.

In conclusion, our study provides new insights into measurement properties of CHIP and the Rasch analyses reported here have added another perspective to the instrument, providing a tentative view of the strengths and weaknesses of its subscales. Two subscales, i.e., revised—“maintaining family integration” and original “maintaining social support”) are unidimensional measurement scales that are efficiently administered and are appropriately targeted for the assessment of coping patterns in parents of children with disabilities. However future studies utilizing larger samples should be undertaken using Rasch analysis to confirm the findings of this study. Broader samples, including a wider variety of children with disabilities drawn from different settings, should be utilized. Further evaluation of the response category format of the instrument should be undertaken to examine the decision made in the study to rescore the categories of the items in the CHIP. Ideally this would include the administration of the original and revised version of the CHIP scoring to the same people to compare their validity. Further research could also examine the longitudinal psychometric properties such as reproducibility and responsiveness to change over time following an intervention (e.g., parental support network, counselling, etc.) of the CHIP. Both the functional subscales are useful outcome measures for large public health studies and in evaluating the impact of interventions, for example, in counseling services for parents of children with disabilities.

## Supporting Information

S1 Data (XLSX)
